# Longitudinal Voice Outcomes After Atelocollagen Injection Laryngoplasty for Vocal Fold Paralysis and Vocal Fold Atrophy/Sulcus

**DOI:** 10.3390/jcm15207772

**Published:** 2026-10-09

**Authors:** Kiyohito Hosokawa, Masayuki Nozawa, Takanari Kawabe, Akihiro Nagata, Itsuki Kitayama, Kenji Aruga, Maki Yamashita, Takeshi Tsuda, Yoshiyuki Ozono, Hidenori Tanaka, Yohei Maeda, Takahito Fukusumi, Makoto Ogawa, Hidenori Inohara

**Affiliations:** 1 Department of Otorhinolaryngology–Head and Neck Surgery, Graduate School of Medicine, The University of Osaka, Suita 565-0871, Osaka, Japan; 2 Department of Otorhinolaryngology, Japan Community Health Care Organization Osaka Hospital, Osaka 553-0003, Japan; 3 Department of Otorhinolaryngology–Head and Neck Surgery, Osaka International Medical & Science Center, Osaka 543-0042, Japan

**Keywords:** atelocollagen, injection laryngoplasty, vocal fold paralysis, vocal fold atrophy, sulcus vocalis, glottic insufficiency, Acoustic Voice Quality Index

## Abstract

**Background:** Atelocollagen injection laryngoplasty is a minimally invasive treatment for glottic insufficiency, but the durability of its effects and whether longitudinal responses differ according to the underlying disorder remain unclear. **Methods:** We retrospectively analyzed prospectively collected clinical data from three institutions. The cohort comprised 92 patients and 142 injection episodes, including 75 patients with vocal fold paralysis and 17 with vocal fold atrophy/sulcus. Acoustic Voice Quality Index (AVQI), maximum phonation time (MPT), and Voice Handicap Index-10 (VHI-10) were evaluated from baseline through 1 year. Longitudinal changes were analyzed using mixed-effects models, and clinically meaningful response was assessed using prespecified criteria. Extended follow-up at 1.5 years and ≥2 years was evaluated in the subset of patients with vocal fold paralysis with available long-term data. **Results:** In vocal fold paralysis, AVQI, MPT, and VHI-10 improved significantly at all follow-up time points through 1 year. In vocal fold atrophy/sulcus, AVQI and VHI-10 showed significant improvement mainly at 1 and 3 months, whereas MPT did not show consistent improvement. The time-by-disease-group interaction was significant for AVQI (*p* = 0.006), but not for MPT (*p* = 0.239) or VHI-10 (*p* = 0.559). AVQI responder trajectories also differed between disease groups (*p* = 0.023). In the available long-term subset (n = 19 at each time point), all three outcomes remained significantly improved at 1.5 years and ≥2 years. At ≥2 years, responder rates were 41.9% for AVQI and 48.4% for VHI-10, and MPT functional recovery was 31.6%. **Conclusions:** Atelocollagen injection laryngoplasty was associated with sustained voice improvement through 1 year in vocal fold paralysis, with improvement also observed at ≥2 years in a selected subset. Effects in vocal fold atrophy/sulcus were less consistently sustained.

## 1. Introduction

Glottic insufficiency resulting from vocal fold paralysis, vocal fold atrophy, or vocal fold sulcus is an important cause of dysphonia. Injection laryngoplasty is a well-established treatment for glottic insufficiency that augments the vocal fold and improves glottic closure by medializing its free edge [1,2]. Advances in injectable materials and endoscopic techniques have broadened the indications for injection laryngoplasty and facilitated its use in awake patients in the office setting, providing a minimally invasive treatment option with lower anesthetic requirements than open laryngeal framework surgery [1,2]. Injection laryngoplasty is therefore widely used for appropriately selected patients with mild-to-moderate glottic insufficiency caused by vocal fold paralysis, paresis, atrophy, scar, or sulcus [1].

Collagen has a long history as an injectable biomaterial for vocal fold augmentation. In a landmark series of 119 patients, Ford et al. demonstrated that injectable bovine collagen could improve glottic insufficiency across a broad spectrum of vocal fold disorders, including atrophy, scarring, and unilateral vocal fold dysfunction, and proposed collagen as a biologic implant capable of integration with host tissue [3]. In Japan, non-crosslinked bovine atelocollagen has been used for vocal fold augmentation for several decades. Kimura et al. reported clinical outcomes in 155 patients treated with 3% non-crosslinked bovine atelocollagen, including patients with unilateral vocal fold paralysis, vocal fold atrophy, and sulcus vocalis, with a mean follow-up of 20 months and follow-up extending to 10 years [4]. Significant improvements in vocal function and perceptual voice quality were observed, particularly in patients with vocal fold paralysis. Histological studies of injectable collagen have demonstrated progressive fibroblast ingrowth and deposition of host collagen within the implant, while experimental studies specifically using atelocollagen have shown retention and accumulation of the injected material within vocal fold tissue [3,5]. These findings provide a possible biological basis for maintenance of functional augmentation despite partial resorption of the injected material.

Despite these favorable clinical experiences, several aspects of the durability of injection laryngoplasty remain incompletely characterized. Clinical benefits after injection with temporary materials may persist beyond their expected period of material resorption, particularly in patients with unilateral vocal fold paralysis [6,7]. Prendes et al. demonstrated sustained improvements in perceptual voice quality and laryngeal findings beyond 6 months after injection with temporary materials, together with a reduced need for subsequent permanent medialization [6], whereas Arviso et al. reported that many patients with potentially recoverable vocal fold paralysis ultimately achieved satisfactory voice through recovery or compensation without permanent surgical intervention [7]. However, systematic longitudinal evidence characterizing how voice outcomes evolve beyond 1 year remains limited, particularly for non-crosslinked atelocollagen, and the durability of treatment effects may differ according to the underlying cause of glottic insufficiency. Although previous studies have included patients with both vocal fold paralysis and vocal fold atrophy or sulcus [3,4], their longitudinal treatment trajectories have not been well characterized using a common analytical framework. Moreover, previous studies have generally evaluated outcomes as group-level changes in acoustic, aerodynamic, perceptual, or patient-reported measures, or according to the need for subsequent intervention [4,6,7]. The proportion of individual patients achieving a prespecified clinically meaningful improvement over time has been less well characterized.

Accordingly, the present multicenter study aimed to characterize the longitudinal effectiveness of atelocollagen injection laryngoplasty in patients with vocal fold paralysis and those with vocal fold atrophy or sulcus. We evaluated changes in acoustic voice quality, phonatory function, and patient-reported voice handicap from baseline through 1 year and examined whether their longitudinal trajectories differed between the two disease groups. We also assessed clinically meaningful individual treatment response using prespecified criteria for each outcome. In addition, extended follow-up at 1.5 years and ≥2 years was examined in the subset of patients with vocal fold paralysis with available long-term data to further characterize outcomes beyond 1 year.

## 2. Materials and Methods

### 2.1. Study Design and Participants

This study was a retrospective analysis of prospectively collected clinical data from patients undergoing atelocollagen injection laryngoplasty at three institutions: Osaka Police Hospital (currently Osaka International Medical & Science Center), Japan Community Health care Organization Osaka Hospital, and The University of Osaka Hospital. During the study period, atelocollagen injection laryngoplasty at these institutions was performed for glottic insufficiency associated with vocal fold paralysis, vocal fold atrophy, or vocal fold sulcus. Voice assessment data collected from October 2015 through 6 August 2026 were included in the database. At the time of data extraction, the registered dataset comprised 166 injection episodes in 114 patients and 612 injection-linked voice-assessment records.

**Figure 1:**
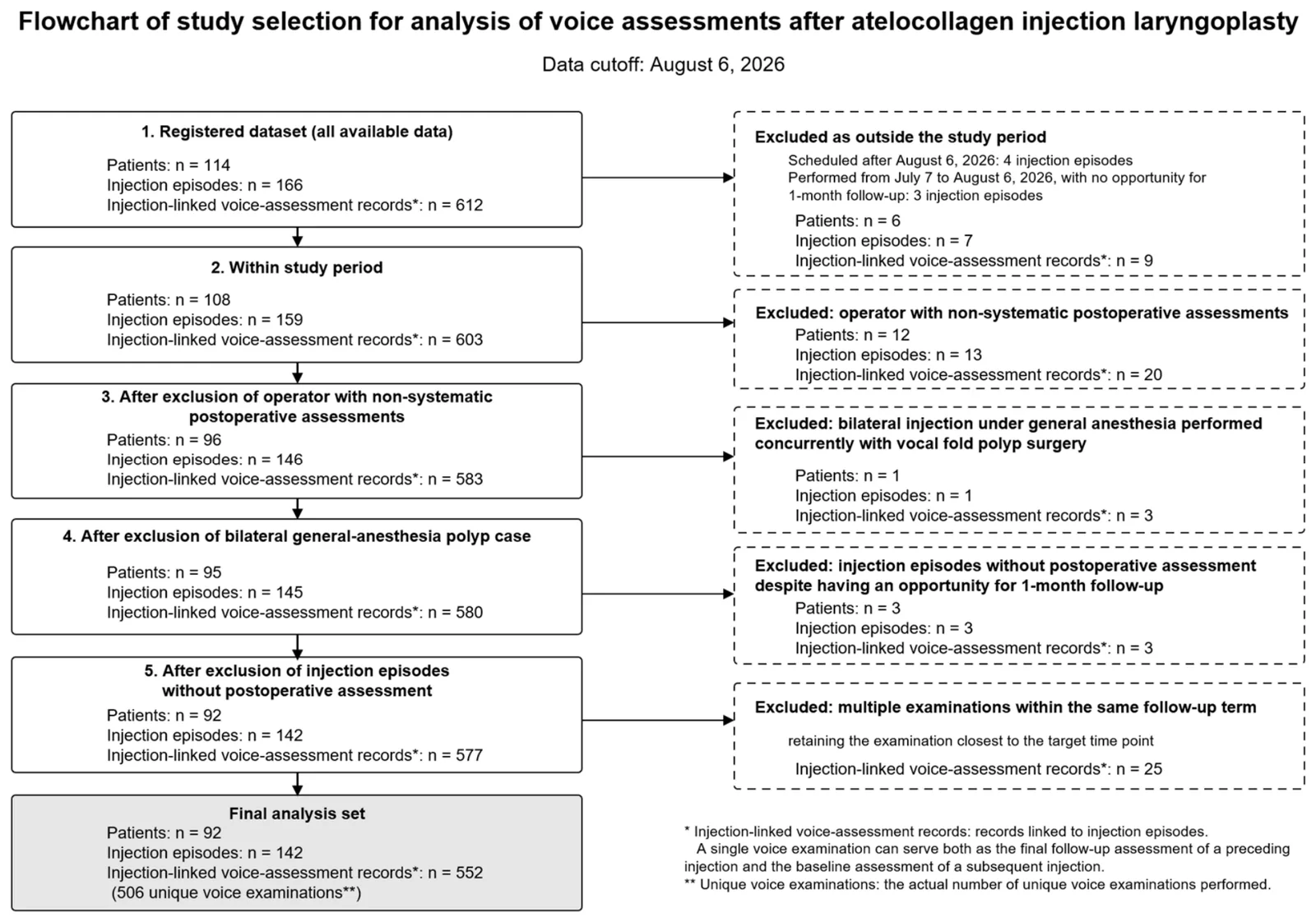
Flowchart of study selection for analysis of voice assessments after atelocollagen injection laryngoplasty.

For the present analysis, seven injection episodes in six patients were excluded because they fell outside the analyzable observation period: four injections were scheduled after 6 August 2026, and three had been performed between 7 July and 6 August 2026, leaving no opportunity for a 1-month follow-up assessment by the data cutoff. This left 159 injection episodes in 108 patients. We then excluded 13 injection episodes in 12 patients treated by an operator for whom postoperative voice assessments had not been systematically obtained, one bilateral injection episode performed under general anesthesia concurrently with vocal fold polyp surgery, and three injection episodes in three patients without any postoperative voice assessment despite adequate opportunity for 1-month follow-up. The final study population comprised 142 injection episodes in 92 patients (Figure 1).

For analysis, vocal fold atrophy and vocal fold sulcus were combined into the Atrophy/Sulcus group, whereas vocal fold paralysis was analyzed separately. Vocal fold atrophy and sulcus vocalis were analyzed together because both represented nonparalytic structural glottic insufficiency treated by augmentation in the present cohort, and the small number of patients with sulcus vocalis precluded meaningful separate longitudinal analysis. Each atelocollagen injection was treated as a distinct injection episode. When a patient underwent multiple injections, each injection constituted a separate episode nested within the same patient. Repeat injections were performed for heterogeneous clinical reasons, including planned additional augmentation in patients with marked glottic insufficiency and reinjection after insufficient or waning benefit. Therefore, the interval between injections was not interpreted as the duration of treatment effect. The most recent pre-injection voice examination was used as the baseline assessment for each episode.

For patients with vocal fold paralysis, the interval from paralysis onset to atelocollagen injection was also examined. To characterize treatment timing in patients in whom spontaneous recovery remained biologically possible, patients with etiologies associated with a very low likelihood of spontaneous recovery, such as documented recurrent laryngeal nerve transection without reconstruction or malignant involvement of the nerve, were excluded from this interval analysis.

The 142 injection episodes were linked to 577 eligible voice-assessment records. When more than one voice examination fell within the same follow-up term for a given injection episode, the examination closest to the target time point was retained. This consolidation removed 25 injection-linked records, resulting in 552 injection-linked records representing 506 unique voice examinations. A single voice examination could serve both as the follow-up assessment of a preceding injection and as the baseline assessment of a subsequent injection.

### 2.2. Atelocollagen Injection Procedure

Atelocollagen injection was performed as an outpatient procedure under topical anesthesia using a double-bent Cathelin needle technique based on the method described by Toyomura et al. [8]. A 60-mm, 23-gauge Cathelin needle was manually bent at two points near its tip to facilitate access to the vocal folds.

The procedure was performed with the patient in the seated position. After topical anesthesia of the laryngopharynx with 4% lidocaine, the larynx was continuously visualized using a transnasal flexible endoscope. The superior thyroid notch was identified by palpation, and the needle was introduced percutaneously approximately 0.5 cm above the notch using a thyrohyoid approach. The needle was advanced through the thyrohyoid membrane, and its tip entered the laryngeal lumen at the base of the epiglottis under endoscopic visualization. The needle tip was then directed toward the target region of the vocal fold.

The injection was targeted to the lateral middle-to-posterior portion of the vocal fold, slightly anterior and lateral to the vocal process. In both vocal fold paralysis and vocal fold atrophy/sulcus, the material was injected into the vocal fold body rather than the superficial cover. Because the bevel of the needle tip faced proximally toward the operator, the needle was directed slightly posterior to the intended injection site to achieve appropriate deposition of the material.

The injectable material was 3% non-crosslinked bovine atelocollagen (Koken Co., Ltd., Tokyo, Japan) [4], supplied in a 1.1-mL syringe. Atelocollagen was injected under direct endoscopic visualization while monitoring vocal fold augmentation. In patients with vocal fold paralysis, injection of nearly the entire 1.1-mL volume was generally attempted; however, the injection was terminated if substantial leakage of the material was observed. In patients with vocal fold atrophy or sulcus, the injected volume was adjusted according to the degree of glottic insufficiency and the augmentation achieved during the procedure. Because variable leakage could occur during injection and the amount of leaked material could not be quantitatively measured, the exact net delivered volume was not available for all procedures.

Voice therapy was not routinely performed before or after injection and was not part of the standardized treatment protocol.

### 2.3. Voice Outcome Measures

Voice outcomes were assessed using the Acoustic Voice Quality Index (AVQI), maximum phonation time (MPT), and Voice Handicap Index-10 (VHI-10).

The AVQI is a multiparametric acoustic measure combining sustained vowel and continuous speech samples and is used as an objective acoustic measure of overall dysphonia severity [9]. Voice samples consisted of a sustained vowel /a/ and connected speech obtained by reading a standardized 30-syllable Japanese passage for AVQI version 03.01 [10,11]. Voice recordings were obtained in a sound-treated room using a head-worn microphone (SE50; Samson Technologies Corp., Hauppauge, NY, USA) and a linear PCM recorder (H4n; Zoom Corp., Tokyo, Japan). The signals were digitized at a sampling rate of 44.1 kHz with 16-bit resolution and stored as WAV files. AVQI was calculated using Praat version 6.2.14 (Paul Boersma and David Weenink, University of Amsterdam, Amsterdam, The Netherlands), with higher values indicating greater dysphonia.

MPT was measured during sustained phonation of the vowel /a/ at the patient’s comfortable pitch and loudness. Three trials were performed, and the longest duration was used for analysis [12].

Voice-related handicap was assessed using the validated Japanese version of the Voice Handicap Index-10 (VHI-10) [13,14,15], with higher scores indicating greater perceived voice-related handicap.

### 2.4. Follow-Up and Analysis Framework

Post-injection voice assessments were performed as part of routine clinical follow-up. Each assessment was assigned to a follow-up term according to the elapsed time from the date of injection: 1 month (>0 to 2 months), 3 months (>2 to 4.5 months), 6 months (>4.5 to 9 months), 1 year (>9 to 15 months), 1.5 years (>15 to 21 months), and ≥2 years (>21 months). The most recent voice assessment before each injection was defined as the baseline assessment. The actual intervals between injection and voice assessment for each follow-up term are summarized in Supplementary Table S1.

The primary analysis evaluated changes from baseline through 1 year (baseline, 1 month, 3 months, 6 months, and 1 year) in both the vocal fold paralysis and Atrophy/Sulcus groups. Longitudinal trajectories of AVQI, MPT, and VHI-10 were evaluated across these time points, together with clinically defined responder or functional recovery rates.

An additional extended follow-up analysis was performed at 1.5 years and ≥2 years in the subset of patients with vocal fold paralysis for whom long-term follow-up data were available. Outcomes at these later time points were evaluated relative to baseline. Because these assessments represented longer-term clinical follow-up rather than uniformly scheduled study visits, the extended analyses were considered complementary to the primary 1-year analysis.

### 2.5. Definitions of Clinical Response

Clinical response was defined separately for AVQI, MPT, and VHI-10.

For AVQI, both the minimal detectable change (MDC) and the minimal important difference (MID) have been reported. An MDC95 of 0.78 has been reported based on test–retest measurements, representing the magnitude of change required to exceed measurement variability [16]. The MID for the Japanese AVQI version 3 has been reported as 0.95 points [17]. Because the present study focused on clinically meaningful improvement after treatment rather than solely on change exceeding measurement variability, the MID was adopted as the responder threshold. Accordingly, an AVQI responder was defined as an injection episode showing a decrease in AVQI of at least 0.95 points from baseline.

For MPT, functional recovery was evaluated using 10 s as the threshold, as an MPT of <10 s has been regarded as abnormally short in adults [12]. Functional recovery was defined as achievement of an MPT of ≥10 s at follow-up among injection episodes with a baseline MPT of <10 s. Injection episodes with a baseline MPT of ≥10 s were therefore excluded from the denominator of the MPT functional recovery analysis.

For VHI-10, clinically meaningful improvement was defined as a decrease of at least 4 points from baseline, based on the minimal clinically important difference reported for patients with unilateral vocal fold paralysis [18]. An injection episode meeting this criterion was classified as a VHI-10 responder. The same threshold was applied to both disease groups to provide a consistent definition of clinically meaningful improvement in the responder analyses.

In the primary responder analyses, subsequent surgical treatment intended to improve glottic closure or voice was considered treatment failure for the preceding atelocollagen injection episode. Observed outcomes obtained before the subsequent surgery were retained, whereas subsequent scheduled follow-up terms were classified as treatment failure. Voice assessments obtained after the subsequent surgery were not attributed to the preceding injection episode, and missing assessments unrelated to subsequent surgery remained missing. An observed-case analysis without classifying subsequent surgery as treatment failure was performed as a sensitivity analysis.

### 2.6. Statistical Analysis

Continuous variables were summarized using medians and interquartile ranges, and categorical variables using counts and percentages. For descriptive within-group comparisons, paired changes from baseline to each follow-up term were assessed using the Wilcoxon signed-rank test. *p* values were adjusted using the Holm method across the four follow-up time points (1 month, 3 months, 6 months, and 1 year) separately within each outcome and disease group. Detailed paired sample sizes and unadjusted *p* values are provided in Supplementary Table S2.

The primary longitudinal analysis included assessments from baseline through 1 year. Linear mixed-effects models were constructed separately for AVQI, MPT, and VHI-10, with assessment term, disease group, and their interaction as fixed effects, together with age and sex. Baseline was included as one of the longitudinal assessment terms and was therefore not entered separately as a baseline covariate. Random intercepts were specified for injection episodes nested within patients to account for repeated assessments within an injection episode and multiple injection episodes within the same patient. AVQI was analyzed on its original scale, whereas MPT was natural-log transformed and VHI-10 was square-root transformed based on model diagnostics. Type III tests with the Kenward–Roger approximation for degrees of freedom were used to evaluate fixed effects. Model-estimated outcomes and 95% confidence intervals were presented on the original scale, with estimates for MPT and VHI-10 back-transformed from the respective transformed scales. Within each disease group, contrasts between baseline and each post-injection term were adjusted for multiple comparisons using the Holm method.

Responder analyses for AVQI and VHI-10 through 1 year were performed using generalized linear mixed-effects models with a binomial distribution and logit link. The models included assessment term, disease group, their interaction, the corresponding baseline outcome value, age, and sex as fixed effects, with the same patient- and injection-level random-effects structure as the longitudinal models. The global significance of the term-by-disease-group interaction was assessed using likelihood-ratio tests comparing models with and without the interaction term. Because the number of eligible injection episodes for MPT functional recovery was limited in the Atrophy/Sulcus group and mixed-model estimation was numerically unstable, MPT functional recovery rates were summarized descriptively without formal mixed-effects model inference.

For the extended follow-up analysis, only injection episodes in the vocal fold paralysis group were evaluated at 1.5 years and ≥2 years. Paired comparisons with baseline were performed using the Wilcoxon signed-rank test, with Holm adjustment across the two extended follow-up comparisons separately for each outcome. Responder and functional recovery rates at these later time points were summarized descriptively.

No imputation was performed for missing continuous outcome data or for missing responder assessments unrelated to subsequent surgery. Mixed-effects models used all available observations. The handling of subsequent voice-improving surgery in the responder analyses followed the treatment-failure definition described above. Observed-case analyses that did not classify subsequent surgery as treatment failure were performed as sensitivity analyses. All tests were two-sided, and *p* < 0.05 was considered statistically significant. Statistical analyses were performed using R version 4.6.0 (R Foundation for Statistical Computing, Vienna, Austria), primarily with the lme4, lmerTest, and emmeans packages.

ChatGPT (GPT-5.6, OpenAI) was used to assist with R code development for statistical analyses and with English-language editing. All generated code and text were reviewed and verified by the authors, who take full responsibility for the analyses and the content of the manuscript.

## 3. Results

### 3.1. Study Population and Follow-Up

The study selection process is shown in Figure 1. Of 166 registered injection episodes in 114 patients, 142 injection episodes in 92 patients met the eligibility criteria and were included in the final analysis. The study population comprised 75 patients with vocal fold paralysis and 17 patients with vocal fold atrophy or sulcus, corresponding to 116 and 26 injection episodes, respectively. After consolidation of multiple examinations within the same follow-up term, 552 injection-linked records representing 506 unique voice examinations were included in the analysis.

Patient characteristics are summarized in Table 1. Among patients with vocal fold paralysis without recurrent laryngeal nerve transection or malignant involvement of the nerve, the median interval from paralysis onset to atelocollagen injection was 461.5 days (IQR, 253.5–1148; n = 61). The median age was 68 years (interquartile range [IQR], 54–75 years; range, 17–88 years), and 58 patients (63.0%) were male. Twenty-nine of 92 patients (31.5%) underwent repeated atelocollagen injections. Among these patients, the number of injections ranged from 2 to 7, yielding 50 consecutive inter-injection intervals, with a median interval of 133 days (IQR, 75–341 days; range, 21–1085 days).

Voice assessments were generally performed close to the predefined target time points. The actual intervals between injection and assessment for each follow-up term are presented in Supplementary Table S1. In the vocal fold paralysis group, the median intervals were 29 days at 1 month, 91 days at 3 months, 182 days at 6 months, and 365 days at 1 year. Corresponding median intervals in the Atrophy/Sulcus group were 30, 91, 183, and 364 days, respectively. In the extended follow-up of the vocal fold paralysis group, median assessment intervals were 546 days at 1.5 years and 734 days at ≥2 years.

One patient developed fever on the day after injection. No other adverse events were documented.

### 3.2. Voice Outcomes and Longitudinal Changes Through 1 Year

Observed voice outcomes from baseline through 1 year are summarized in Table 2, with detailed paired comparisons provided in Supplementary Table S2. In the vocal fold paralysis group, median AVQI decreased from 5.10 (IQR, 3.07–7.75) at baseline to 3.40 (2.05–5.92) at 1 month, 3.07 (1.57–5.50) at 3 months, 2.27 (1.35–3.98) at 6 months, and 2.51 (1.27–4.00) at 1 year. Median MPT increased from 8.02 s (5.48–11.80) at baseline to 9.80 s (7.07–14.94), 9.89 s (6.92–14.22), 11.46 s (7.11–16.68), and 11.40 s (8.00–16.27), respectively, while median VHI-10 decreased from 18.0 (11.0–28.0) to 11.0 (3.0–21.0), 13.0 (3.0–20.0), 7.5 (3.0–16.2), and 4.5 (2.2–13.0), respectively. Paired comparisons with baseline demonstrated significant improvement at every follow-up time point for all three outcomes after Holm adjustment (all adjusted *p* < 0.001, except MPT at 6 months, *p* = 0.001).

**Figure 2:**
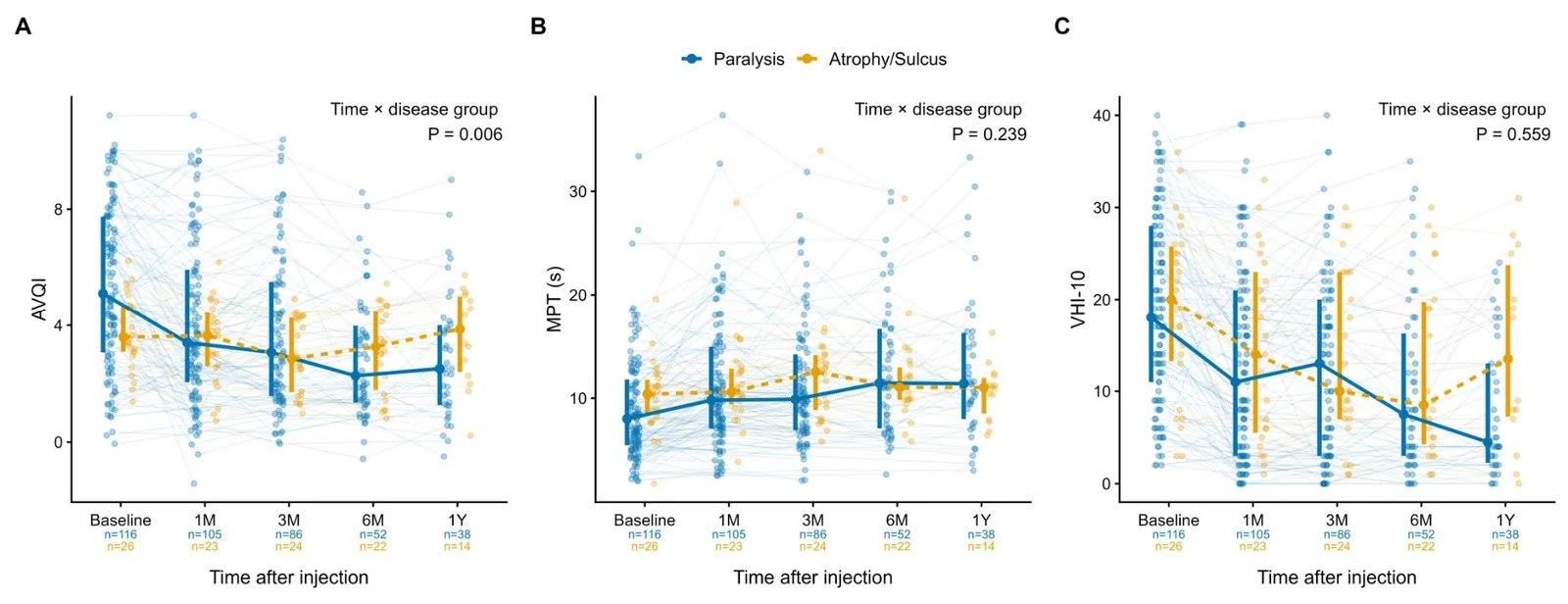
Longitudinal voice outcomes through 1 year after atelocollagen injection laryngoplasty. Individual observations and within-injection trajectories are shown for vocal fold paralysis and vocal fold atrophy/sulcus. Large circles and vertical bars indicate medians and interquartile ranges, respectively; numbers below each time point indicate available injection episodes. *p* values indicate the time-by-disease-group interaction from the linear mixed-effects models. (**A**) AVQI; (**B**) MPT; (**C**) VHI-10.

In the Atrophy/Sulcus group, median AVQI was 3.59 (3.09–4.64) at baseline, 3.64 (2.59–4.44) at 1 month, 2.85 (1.70–4.27) at 3 months, 3.26 (1.78–4.48) at 6 months, and 3.87 (2.40–5.00) at 1 year. Paired comparisons showed significant improvement at 1 month and 3 months (adjusted *p* = 0.028 and 0.009, respectively), but not at 6 months or 1 year. MPT did not differ significantly from baseline at any follow-up time point. VHI-10 decreased from a median of 20.0 (13.2–25.8) at baseline to 14.0 (5.5–23.0) at 1 month and 10.0 (7.0–23.0) at 3 months, with significant paired improvements at these two early time points (adjusted *p* = 0.008 and 0.016, respectively), whereas differences at 6 months and 1 year were not significant. The observed longitudinal distributions and within-episode trajectories of the three voice outcomes are shown in Figure 2. Full fixed-effect coefficients and model-estimated outcomes are provided in Supplementary Tables S3 and S4, respectively.

Longitudinal mixed-effects model results are summarized in Table 3. Significant overall effects of time were observed for AVQI, MPT, and VHI-10 (all *p* < 0.001). Importantly, the time-by-disease-group interaction was significant for AVQI (*p* = 0.006), indicating that the longitudinal trajectory of acoustic voice quality differed between the vocal fold paralysis and Atrophy/Sulcus groups. The adjusted model-estimated AVQI in the vocal fold paralysis group decreased from 5.10 (95% CI, 4.58–5.63) at baseline to 3.71 (3.18–4.24) at 1 month and remained lower at 1 year at 3.56 (2.92–4.19). In the Atrophy/Sulcus group, the corresponding estimates were 2.92 (1.75–4.10) at baseline, 2.51 (1.32–3.70) at 1 month, and 2.87 (1.58–4.16) at 1 year. In contrast, no significant time-by-disease-group interactions were observed for MPT (*p* = 0.239) or VHI-10 (*p* = 0.559).

### 3.3. Clinical Response Through 1 Year

Clinical response rates through 1 year are summarized in Table 4. In the vocal fold paralysis group, AVQI responder rates were 57.1% (60/105) at 1 month, 43.7% (38/87) at 3 months, 46.6% (27/58) at 6 months, and 40.8% (20/49) at 1 year. Corresponding rates in the Atrophy/Sulcus group were 26.1% (6/23), 45.8% (11/24), 34.8% (8/23), and 20.0% (3/15), respectively. In the generalized linear mixed-effects model, the trajectory of AVQI response differed significantly between disease groups (term-by-disease-group interaction, *p* = 0.023).

Among injection episodes with a baseline MPT of <10 s, functional recovery to an MPT of ≥10 s was observed in 27.4% (20/73), 26.8% (15/56), 37.1% (13/35), and 32.3% (10/31) of episodes in the vocal fold paralysis group at 1 month, 3 months, 6 months, and 1 year, respectively. The corresponding rates in the Atrophy/Sulcus group were 50.0% (5/10), 45.5% (5/11), 36.4% (4/11), and 33.3% (2/6). Because of the limited number of eligible episodes, particularly in the Atrophy/Sulcus group, MPT functional recovery was evaluated descriptively without formal mixed-effects model inference.

For VHI-10, responder rates in the vocal fold paralysis group were 60.0% (63/105) at 1 month, 50.6% (44/87) at 3 months, 62.1% (36/58) at 6 months, and 55.1% (27/49) at 1 year. Corresponding rates in the Atrophy/Sulcus group were 52.2% (12/23), 45.8% (11/24), 43.5% (10/23), and 33.3% (5/15), respectively. The term-by-disease-group interaction was not significant for VHI-10 response (*p* = 0.737). Observed-case responder and functional recovery rates are provided in Supplementary Table S5.

### 3.4. Extended Follow-Up in Vocal Fold Paralysis

Given the sustained improvement observed through 1 year in the vocal fold paralysis group, extended follow-up outcomes at 1.5 years and ≥2 years were evaluated in the subset with available long-term data (Table 5). Paired long-term assessments were available for 19 injection episodes at each time point; therefore, these analyses describe observed outcomes in the available long-term subset.

Among 19 injection episodes with paired baseline and 1.5-year assessments, median AVQI decreased from 4.26 (IQR, 3.06–6.03) to 3.11 (1.36–4.03), median MPT increased from 8.49 s (6.34–11.3) to 11.5 s (8.11–13.2), and median VHI-10 decreased from 10.0 (5.5–15.5) to 3.0 (1.5–13.5). All three changes were significant after Holm adjustment (AVQI, *p* = 0.003; MPT, *p* = 0.008; VHI-10, *p* = 0.010).

Similarly, among 19 injection episodes with paired baseline and ≥2-year assessments, median AVQI decreased from 4.25 (2.25–4.79) to 2.19 (1.26–3.08), median MPT increased from 8.98 s (7.54–12.5) to 13.5 s (10.6–15.8), and median VHI-10 decreased from 12.0 (7.5–15.5) to 4.0 (2.0–7.0). These changes were also significant after Holm adjustment (AVQI, *p* = 0.008; MPT, *p* = 0.008; VHI-10, *p* < 0.001).

Using the primary responder definition in which subsequent voice-improving surgery was considered treatment failure, AVQI responder rates were 36.7% (11/30) at 1.5 years and 41.9% (13/31) at ≥2 years. Corresponding VHI-10 responder rates were 36.7% (11/30) and 48.4% (15/31), respectively. Among injection episodes with a baseline MPT < 10 s, functional recovery rates were 20.0% (4/20) at 1.5 years and 31.6% (6/19) at ≥2 years.

## 4. Discussion

The present multicenter study demonstrated distinct longitudinal patterns of voice outcomes after atelocollagen injection laryngoplasty according to the underlying cause of glottic insufficiency. In patients with vocal fold paralysis, significant improvements in AVQI, MPT, and VHI-10 were consistently observed through 1 year after injection. In contrast, patients with vocal fold atrophy or sulcus showed significant improvement mainly during the early follow-up period, without clear evidence of sustained improvement through 1 year. The longitudinal mixed-effects analysis further demonstrated a significant time-by-disease-group interaction for AVQI, indicating that the trajectory of acoustic voice quality differed between the two disease groups, whereas no corresponding interaction was observed for MPT or VHI-10. Clinically meaningful responder analyses showed a similar disease-related pattern for AVQI. Furthermore, among patients with vocal fold paralysis, significant improvements in all three voice outcomes were observed at 1.5 years and ≥2 years in the available long-term follow-up subset, indicating that prolonged benefit can occur in some patients.

The sustained improvement observed in vocal fold paralysis is consistent with previous reports indicating that the clinical effects of injection laryngoplasty can extend well beyond the expected persistence of the injected material. Using the same 3% non-crosslinked bovine atelocollagen, Kimura et al. reported persistent improvements in vocal function in patients followed for at least 2 years [4]. Long-term benefit has also been demonstrated with other collagen-based or temporary injectable materials. In a prospective study comparing cross-linked hyaluronan with bovine cross-linked collagen, Hertegård et al. reported sustained improvements in patient-rated voice and glottal closure at 24 months, although approximately one-quarter of patients underwent repeat injection or medialization surgery [19]. More recently, Yun et al. reported that, among patients with unilateral vocal fold paralysis treated with injection laryngoplasty, the estimated persistence of treatment effect was 63.4% at 1 year and 59.4% at 2 years [20]. Histological studies have demonstrated fibroblast ingrowth and host collagen deposition within injectable collagen implants [3], while Matsui et al. reported retention and accumulation of atelocollagen within vocal fold tissue after repeated injection [5]. These histological findings provide biological plausibility for prolonged augmentation; however, the present study was not designed to determine whether material persistence or tissue integration accounted for the sustained clinical improvement. The timing of treatment in the present cohort also makes spontaneous neural recovery less likely to be the principal explanation for the observed improvement. Among patients with vocal paralysis in whom spontaneous recovery remained biologically possible, the median interval from paralysis onset to injection was 461.5 days (IQR, 253.5–1148 days). Nevertheless, in the absence of an untreated control group, spontaneous recovery and natural compensatory changes cannot be completely excluded.

The difference in longitudinal response between vocal fold paralysis and vocal fold atrophy/sulcus was another important finding of the present study. In vocal fold paralysis, improvements in AVQI, MPT, and VHI-10 were maintained through 1 year, whereas in vocal fold atrophy/sulcus, significant improvements were observed mainly at 1 and 3 months and were less evident thereafter. This difference was most clearly demonstrated for AVQI, for which the time-by-disease-group interaction was significant. A similar tendency was reported by Kimura et al., who found more consistent improvement after atelocollagen injection in vocal fold paralysis than in vocal fold atrophy or sulcus [4]. Outcomes with other injectable materials also suggest that the response to augmentation may depend on the underlying disorder. In a multicenter study of carboxymethylcellulose injection, Mallur et al. reported significant VHI-10 improvement particularly in patients with vocal fold immobility and hypomobility, although patients with atrophy and scar/sulcus were also treated [21]. More recently, Venkatraman et al. demonstrated considerable variability in treatment durability among patients undergoing injection augmentation for presbyphonia; 40% underwent repeat injection, and durability was influenced by injectable material and adjunctive voice therapy [22]. The different responses may reflect differences in the underlying pathophysiology of glottic insufficiency. In unilateral vocal fold paralysis, augmentation of a relatively localized glottic gap can directly improve medialization and phonatory efficiency, whereas vocal fold atrophy and sulcus are often associated with diffuse loss of vocal fold volume, altered tissue properties, or impaired vibratory characteristics that cannot be fully corrected by augmentation alone [1,4]. These findings suggest that atelocollagen injection may provide a more predictable and sustained benefit for vocal fold paralysis, while its effects in vocal fold atrophy/sulcus may be more variable and less durable.

In contrast to traditional voice measures commonly used to evaluate the effects of injection laryngoplasty, the AVQI adopted in the present study provides several complementary benchmarks that facilitate interpretation of treatment-related change. As a multiparametric measure incorporating both sustained vowel and continuous speech, AVQI provides an objective estimate of overall dysphonia severity [9,10,11]. Importantly, its results can be interpreted using distinct reference values: a diagnostic cutoff indicates whether voice quality falls within the normal range, the MDC indicates whether an observed change exceeds expected measurement variability, and the MID indicates whether the magnitude of change is clinically meaningful [11,16,17]. In the present study, the MID of 0.95 points was used to define AVQI response rather than normalization to the diagnostic cutoff, because crossing the normality threshold alone does not necessarily represent a change exceeding measurement variability. The availability of these complementary interpretive benchmarks makes AVQI particularly useful not only for evaluating treatment efficacy in clinical research but also for communicating treatment outcomes in clinical practice. By translating an acoustic measurement into clinically interpretable concepts such as normality, reliable change, and meaningful improvement, AVQI may facilitate a shared understanding of treatment outcomes between clinicians and patients.

The present findings also have practical implications for the clinical use of atelocollagen injection laryngoplasty. In patients with vocal fold paralysis, the possibility of sustained benefit through and beyond 1 year suggests that atelocollagen injection should not necessarily be regarded simply as a short-term or temporary intervention. Injection laryngoplasty also occupies a flexible position within the treatment pathway for unilateral vocal fold paralysis. Comparative studies have shown improvements in subjective, perceptual, and laryngoscopic voice outcomes after both injection laryngoplasty and medialization laryngoplasty, without clear differences in the magnitude of improvement between the two approaches [23,24]. Laryngeal reinnervation represents another treatment option for unilateral vocal fold paralysis, particularly when restoration of longer-term neuromuscular function is desired. Recent evidence, including a systematic review and meta-analysis by Guarino et al., supports favorable functional outcomes after reinnervation, although patient selection, timing, and surgical expertise remain important considerations [25]. The minimally invasive and repeatable nature of injection laryngoplasty therefore allows treatment to be tailored according to the clinical course while preserving the option of subsequent framework surgery when adequate benefit is not achieved or maintained. At the same time, the more variable course observed in vocal fold atrophy/sulcus suggests that expectations regarding durability should be individualized according to the underlying disorder. Thus, longitudinal assessment after injection is important not only for determining whether initial improvement has occurred but also for deciding whether observation, repeat injection, or transition to another treatment strategy is appropriate.

Several limitations should be acknowledged. First, this was a retrospective analysis of routinely collected clinical data, and the timing and availability of postoperative assessments were therefore not uniform. Although the mixed-effects models allowed the use of unbalanced longitudinal data, later follow-up was available for only a subset of patients, particularly at 1.5 years and ≥2 years. The extended follow-up findings are therefore susceptible to selection and survivorship bias and should be interpreted as evidence that prolonged benefit can occur rather than as an estimate of the probability of maintaining benefit beyond 1 year. Second, the vocal fold atrophy/sulcus group was relatively small and included two related but distinct conditions, limiting statistical power and precluding separate analyses of atrophy and sulcus. The smaller number of patients in this group also reflects treatment selection in routine clinical practice; based on accumulated clinical experience that the benefit of atelocollagen injection was less consistent in vocal fold atrophy and sulcus than in vocal fold paralysis, injection was not routinely recommended as actively for these conditions. This clinical selection may have introduced indication bias and limits the generalizability of the findings for vocal fold atrophy/sulcus. Third, some patients underwent multiple injections, and the clinical reasons for repeat injection were heterogeneous, including planned additional augmentation and reinjection after insufficient or waning benefit. Accordingly, the interval between injections should not be interpreted as the duration of treatment effect. Although each injection was treated as a separate episode nested within the patient in the longitudinal analyses, cumulative effects of previous injections or tissue remodeling cannot be entirely excluded. In addition, the exact net delivered volume could not be quantified in all procedures because leakage during injection was not measurable. Voice therapy was not routinely performed as part of the treatment protocol, and occasional use was not systematically captured. Finally, the study did not include an untreated or alternative-treatment control group; therefore, the observed longitudinal changes cannot be attributed exclusively to atelocollagen injection. Prospective studies with standardized follow-up schedules and larger disease-specific cohorts would help further define treatment durability and identify patients most likely to achieve sustained benefit.

## 5. Conclusions

Atelocollagen injection laryngoplasty was associated with sustained improvement in acoustic voice quality, phonatory function, and voice-related handicap through 1 year in patients with vocal fold paralysis. In contrast, improvement in vocal fold atrophy/sulcus was more evident during the early post-injection period and was less consistently maintained over time, with a significant difference in the longitudinal AVQI trajectory between disease groups. Clinically meaningful responder analyses supported these findings at the individual level. In the subset of patients with vocal fold paralysis who had available extended follow-up, significant improvements were also observed at 1.5 years and ≥2 years, indicating that prolonged benefit can occur in some patients. These findings support atelocollagen injection as a minimally invasive treatment option for vocal fold paralysis, while emphasizing the need for individualized expectations and longitudinal assessment in vocal fold atrophy/sulcus.

## Figures and Tables

**Table 1:** Patient characteristics.

Characteristic	Value
**Patients, n**	92
**Sex, n (%)**	
Male	58 (63.0%)
Female	34 (37.0%)
**Age, years**	
Median (IQR)	68 (54–75)
Range	17–88
**Diagnosis, n (%)**	
Vocal fold paralysis	75 (81.5%)
Vocal fold atrophy/sulcus vocalis	17 (18.5%)
Vocal fold atrophy	13 (14.1%)
Sulcus vocalis	4 (4.3%)
Interval from paralysis onset to injection, days *	
Median (IQR)	461.5 (253.5–1148)
Patients included in interval analysis, n	61
**Etiology of vocal fold paralysis, n (% of patients with paralysis)**	
Idiopathic	21 (28.0%)
Thyroid surgery	12 (16.0%)
Thoracic aortic surgery	11 (14.7%)
Esophageal cancer surgery	9 (12.0%)
Thymic/mediastinal surgery	6 (8.0%)
Lung/pulmonary surgery	5 (6.7%)
Cervical spine surgery	3 (4.0%)
Malignant nerve involvement	3 (4.0%)
Vagal/cervical schwannoma surgery	2 (2.7%)
Jugular foramen syndrome	2 (2.7%)
Cardiac surgery	1 (1.3%)

* Calculated among patients with vocal fold paralysis in whom spontaneous recovery remained biologically possible; patients with recurrent laryngeal nerve transection without reconstruction or malignant involvement of the nerve were excluded.

**Table 2:** Voice outcomes through 1 year after atelocollagen injection.

Outcome	Disease Group	Baseline	1 Month	*p*	3 Months	*p*	6 Months	*p*	1 Year	*p*
AVQI	Paralysis	5.10 (3.07–7.75), n = 116	**3.40 (2.05–5.92), n = 105**	**<0.001**	**3.07 (1.57–5.50), n = 86**	**<0.001**	**2.27 (1.35–3.98), n = 52**	**<0.001**	**2.51 (1.27–4.00), n = 38**	**<0.001**
AVQI	Atrophy/Sulcus	3.59 (3.09–4.64), n = 26	**3.64 (2.59–4.44), n = 23**	**0.028**	**2.85 (1.70–4.27), n = 24**	**0.009**	3.26 (1.78–4.48), n = 22	0.288	3.87 (2.40–5.00), n = 14	0.379
MPT	Paralysis	8.02 (5.48–11.80), n = 116	**9.80 (7.07–14.94), n = 105**	**<0.001**	**9.89 (6.92–14.22), n = 86**	**<0.001**	**11.46 (7.11–16.68), n = 52**	**0.001**	**11.40 (8.00–16.27), n = 38**	**<0.001**
MPT	Atrophy/Sulcus	10.39 (8.33–11.77), n = 26	10.60 (9.38–12.84), n = 23	0.149	12.52 (8.85–14.14), n = 24	0.087	11.04 (9.86–12.95), n = 22	0.183	11.02 (8.52–11.95), n = 14	0.950
VHI-10	Paralysis	18.0 (11.0–28.0), n = 116	**11.0 (3.0–21.0), n = 105**	**<0.001**	**13.0 (3.0–20.0), n = 86**	**<0.001**	**7.5 (3.0–16.2), n = 52**	**<0.001**	**4.5 (2.2–13.0), n = 38**	**<0.001**
VHI-10	Atrophy/Sulcus	20.0 (13.2–25.8), n = 26	**14.0 (5.5–23.0), n = 23**	**0.008**	**10.0 (7.0–23.0), n = 24**	**0.016**	8.5 (4.2–19.8), n = 22	0.104	13.5 (7.2–23.8), n = 14	0.195

Values are presented as median (interquartile range) and number of available observations. *p* values represent comparisons with baseline using paired Wilcoxon signed-rank tests and were adjusted using the Holm method across the four follow-up time points (1 month, 3 months, 6 months, and 1 year) separately for each outcome and disease group. Detailed paired sample sizes and unadjusted *p* values are provided in Supplementary Table S2. Bold values indicate adjusted *p* < 0.05. AVQI, Acoustic Voice Quality Index; MPT, maximum phonation time; VHI-10, Voice Handicap Index-10.

**Table 3:** Global tests of fixed effects from longitudinal mixed-effects models of voice outcomes through 1 year after atelocollagen injection.

Outcome	Time	Disease Group	Time × Disease Group	Age	Sex
AVQI	**<0.001**	**0.028**	**0.006**	0.178	**0.001**
MPT	**<0.001**	0.246	0.239	**0.004**	0.215
VHI-10	**<0.001**	0.863	0.559	0.097	0.106

Values are *p* values from Type III tests of fixed effects using the Kenward–Roger approximation for degrees of freedom. The longitudinal analysis included baseline, 1 month, 3 months, 6 months, and 1 year as repeated outcome time points. Each model included time, disease group, the time-by-disease group interaction, age, and sex as fixed effects, with random intercepts for patients and injection episodes nested within patients. Because baseline was included as an outcome time point, the baseline value of the corresponding outcome was not entered as a covariate. AVQI was analyzed on the original scale, MPT after natural-log transformation, and VHI-10 after square-root transformation based on model diagnostics. Bold values indicate *p* < 0.05. AVQI, Acoustic Voice Quality Index; MPT, maximum phonation time; VHI-10, Voice Handicap Index-10.

**Table 4:** Responder and functional recovery rates through 1 year after atelocollagen injection.

Outcome	Time	Paralysis	Atrophy/Sulcus	*p* for Time × Disease-Group Interaction
**AVQI responder**	1 month	60/105 (57.1%)	6/23 (26.1%)	**0.023**
	3 months	38/87 (43.7%)	11/24 (45.8%)	
	6 months	27/58 (46.6%)	8/23 (34.8%)	
	1 year	20/49 (40.8%)	3/15 (20.0%)	
**VHI-10 responder**	1 month	63/105 (60.0%)	12/23 (52.2%)	0.737
	3 months	44/87 (50.6%)	11/24 (45.8%)	
	6 months	36/58 (62.1%)	10/23 (43.5%)	
	1 year	27/49 (55.1%)	5/15 (33.3%)	
**MPT functional recovery**	1 month	20/73 (27.4%)	5/10 (50.0%)	Not performed
	3 months	15/56 (26.8%)	5/11 (45.5%)	
	6 months	13/35 (37.1%)	4/11 (36.4%)	
	1 year	10/31 (32.3%)	2/6 (33.3%)	

Values are presented as n/N (%). An AVQI responder was defined as a decrease of ≥0.95 points from baseline, and a VHI-10 responder as a decrease of ≥4 points from baseline. MPT functional recovery was defined as achievement of an MPT ≥ 10 s among injection episodes with a baseline MPT < 10 s. Subsequent voice-improving surgery was considered treatment failure: measurements obtained before subsequent surgery were evaluated according to the observed outcome, whereas scheduled assessment periods after the last preoperative assessment were classified as treatment failures. Missing assessments unrelated to subsequent surgery were treated as missing. Accordingly, denominators may exceed the number of observed voice assessments because episodes undergoing subsequent surgery remained evaluable as treatment failures. Time-by-disease group interactions for AVQI and VHI-10 responder status were evaluated using generalized linear mixed-effects models adjusted for the baseline value of the corresponding outcome, age, and sex, with random intercepts for patients and injection episodes nested within patients. *p* values were obtained using likelihood-ratio tests comparing models with and without the interaction term. Formal between-group inference for MPT functional recovery was not performed because of the limited number of eligible injection episodes in the atrophy/sulcus group and numerical instability of the generalized linear mixed-effects model. AVQI, Acoustic Voice Quality Index; MPT, maximum phonation time; VHI-10, Voice Handicap Index-10. Bold values indicate *p* < 0.05.

**Table 5:** Extended follow-up outcomes in vocal fold paralysis.

(**A**) Paired long-term voice outcomes							
**Outcome**	**Follow-Up**		**Paired n**	**Baseline**		**Follow-Up**	**Holm-Adjusted *p***
**AVQI**	1.5 years		19	4.26 (3.06–6.03)		3.11 (1.36–4.03)	**0.003**
	≥2 years		19	4.25 (2.25–4.79)		2.19 (1.26–3.08)	**0.008**
**MPT, s**	1.5 years		19	8.49 (6.34–11.3)		11.5 (8.11–13.2)	**0.008**
	≥2 years		19	8.98 (7.54–12.5)		13.5 (10.6–15.8)	**0.008**
**VHI-10**	1.5 years		19	10.0 (5.5–15.5)		3.0 (1.5–13.5)	**0.010**
	≥2 years		19	12.0 (7.5–15.5)		4.0 (2.0–7.0)	**<0.001**
(**B**) Long-term responder and functional recovery rates							
**Outcome**		**1.5 Years**			**≥2 Years**		
AVQI responder		11/30 (36.7%)			13/31 (41.9%)		
VHI-10 responder		11/30 (36.7%)			15/31 (48.4%)		
MPT functional recovery		4/20 (20.0%)			6/19 (31.6%)		

(**A**) values are median (interquartile range) from injection episodes with paired baseline and long-term follow-up measurements. *p* values were calculated using paired Wilcoxon signed-rank tests and adjusted using the Holm method across the two long-term comparisons (1.5 years and ≥2 years) separately for each outcome. Panel B values are n/N (%). An AVQI responder was defined as a decrease of ≥0.95 points from baseline, and a VHI-10 responder as a decrease of ≥4 points from baseline. MPT functional recovery was defined as achievement of an MPT ≥ 10 s among injection episodes with a baseline MPT < 10 s. For (**B**), subsequent voice-improving surgery was considered treatment failure: measurements obtained before subsequent surgery were evaluated according to the observed outcome, whereas scheduled assessment periods after the last preoperative assessment were classified as treatment failures. Missing assessments unrelated to subsequent surgery were treated as missing. Thus, denominators in Panel B may exceed the number of observed long-term voice assessments. AVQI, Acoustic Voice Quality Index; MPT, maximum phonation time; VHI-10, Voice Handicap Index-10. Bold values indicate adjusted *p* < 0.05.

## Data Availability

The data supporting the findings of this study are presented in the article and its Supplementary Materials. Individual-level clinical data and original voice recordings are not publicly available because of privacy and ethical restrictions. The R code used for the statistical analyses may be made available by the corresponding author upon reasonable request.

## Supplementary Materials

The following supporting information can be downloaded at: https://www.mdpi.com/article/10.3390/jcm15207772/s1, Supplementary Table S1: Actual timing of voice assessments according to follow-up term; Supplementary Table S2: Detailed paired comparisons of voice outcomes from baseline through 1 year after atelocollagen injection; Supplementary Table S3: Fixed-effect coefficients from longitudinal mixed-effects models of voice outcomes through 1 year after atelocollagen injection; Supplementary Table S4: Model-estimated voice outcomes through 1 year after atelocollagen injection; Supplementary Table S5: Observed-case responder and functional recovery rates through 1 year after atelocollagen injection.
